# Supplementary material for: Fluctuations in instantaneous frequency predict alpha amplitude during visual perception
Source: Nat Commun. 2017 Dec 12;8:2071. doi: 10.1038/s41467-017-02176-x (PMC5727061; doi:10.1038/s41467-017-02176-x)
Supplement: Supplementary file 1 — Supplementary Information [file 41467_2017_2176_MOESM1_ESM.pdf]

Supplementary Information

Supplementary Methods: Harmonic Oscillators - Amplitude and frequency are linked through oscillatory drive and local dampening

Here we derive an expression for amplitude in a target region (A_T) as a function of parameters of oscillators in the target and driving regions. We model voltage changes in time v(t) in the target region as a damped, driven harmonic oscillator with a second order differential equation of the form:

(1) $mv(t)'' + cv(t)' + kv(t) = D(t)$

Where $I(t)$ is sinusoidal driving force, let $D(t) = A_D\cos(\omega_D t)$ without loss of generality (using sin to model the driving force yields identical results). The characteristic frequency in this target region (i.e. absent of driving force $D(t)$) depends on the parameters of this oscillator:

(2) $\omega_T = \sqrt{k/m}$

Using the method of undetermined coefficients, the steady state (particular) solution of (1) is

(3) $v_{ss} = a\cos(\omega_D t) + b\sin(\omega_D t)$

Where amplitude in the target region (A_T) depends on coefficients a and b as follows

(4) $A_T = \sqrt{a^2 + b^2}$

To find expressions for a and b, and thus for A_T, we substitute v_{ss} in (2) for v in (1) to obtain

(5) $(-am\omega_D{}^2 + bc\omega_D + ak)\cos(\omega_D t) + (-bm\omega_D{}^2 - ac\omega_D + bk)\sin(\omega_D t) = A_D\cos(\omega_D t)$

Equating terms with sin and cos yields the following system of equations:

(6) $a(k - m\omega_D{}^2) + bc\omega_D = A_D$
$b(k - m\omega_D{}^2) - ac\omega_D = 0$

Solving this system for a and b, we obtain

(7) $a(k - m\omega_D{}^2)^2 + ac^2\omega_D{}^2 = A_D(k - m\omega_D{}^2)$

$$a = A_D \frac{k - m\omega_D{}^2}{(k - m\omega_D{}^2)^2 + c^2\omega_D{}^2}$$

$b(k - m\omega_D{}^2)^2 + bc^2\omega_D{}^2 = A_D c\omega_D$

$$b = A_D \frac{c\omega_D}{(k - m\omega_D{}^2)^2 + c^2\omega_D{}^2}$$

Given $(k - m\omega_D{}^2)^2 + \omega_D{}^2 c^2 \neq 0$. We then utilize the identity in (2) to write the system of equations in terms of ω_T

(4) $a = A_D \dfrac{m(\omega_T{}^2 - \omega_D{}^2)}{m^2(\omega_T{}^2 - \omega_D{}^2)^2 + c^2\omega_D{}^2}$

$$b = A_D \frac{c\,\omega_D}{m^2(\omega_T{}^2 - \omega_D{}^2)^2 + c^2\,\omega_D{}^2}$$

Finally, we can combine a and b using (4) to show that

$$(5)\ A_T = \sqrt{a^2 + b^2} = \frac{A_D}{\sqrt{m^2(\omega_T{}^2 - \omega_D{}^2)^2 + c^2\,\omega_D{}^2}}$$

This demonstrates that A_T depends directly on ω_T. If there is no dampening, i.e. $c = 0$, maximal A_T is achieved as $\omega_D \rightarrow \omega_T$ (note that without dampening, A_T is infinite when $\omega_D = \omega_T$). In the case of dampening, $c > 0$, the effective characteristic frequency in the target region, ω_{eT} is bounded as follows: $\omega_{eT} \leq \sqrt{\omega_T{}^2 - \frac{c^2}{2m^2}}$ and maximal A_T is achieved as $\omega_D \rightarrow \omega_{eT}$. In both cases, amplitude in the target region will be maximal when that region is receiving ω_D close to its intrinsic frequency, and will fall off as $|\omega_D - \omega_{(e)T}|$ grows. This demonstrates that there are inherent dependencies between amplitude and frequency using the simplest possible model of a driven oscillation.

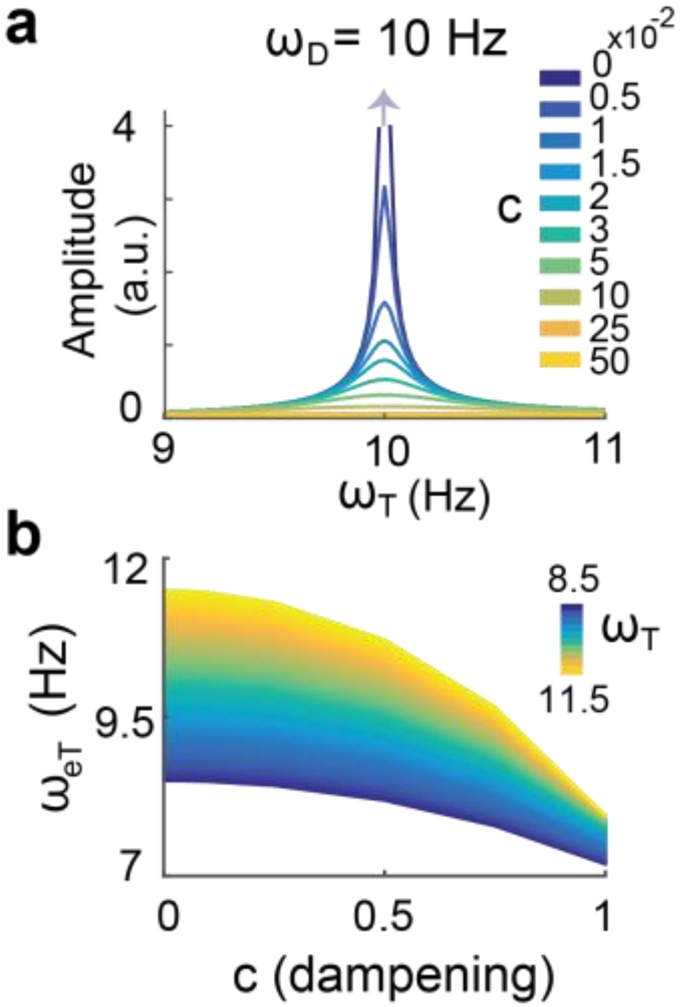

Supplementary Figure 1: Oscillatory Drive and dampening link amplitude and frequency
a) When being driven at 10 Hz, amplitude in the target region is determined by the local frequency for a range of damping values (indicated by the colored lines and legend). With zero dampening (purple line), and frequency in the target region = 10 Hz, runaway resonance occurs (i.e. amplitude approaches infinity), as indicated by the purple arrow. **b)** This dampening that prevents infinite amplitude also effects the effective frequency in the target region, further determining where the target region falls on the x-axis of panel A. With more damping, the effective frequency in the target region falls.

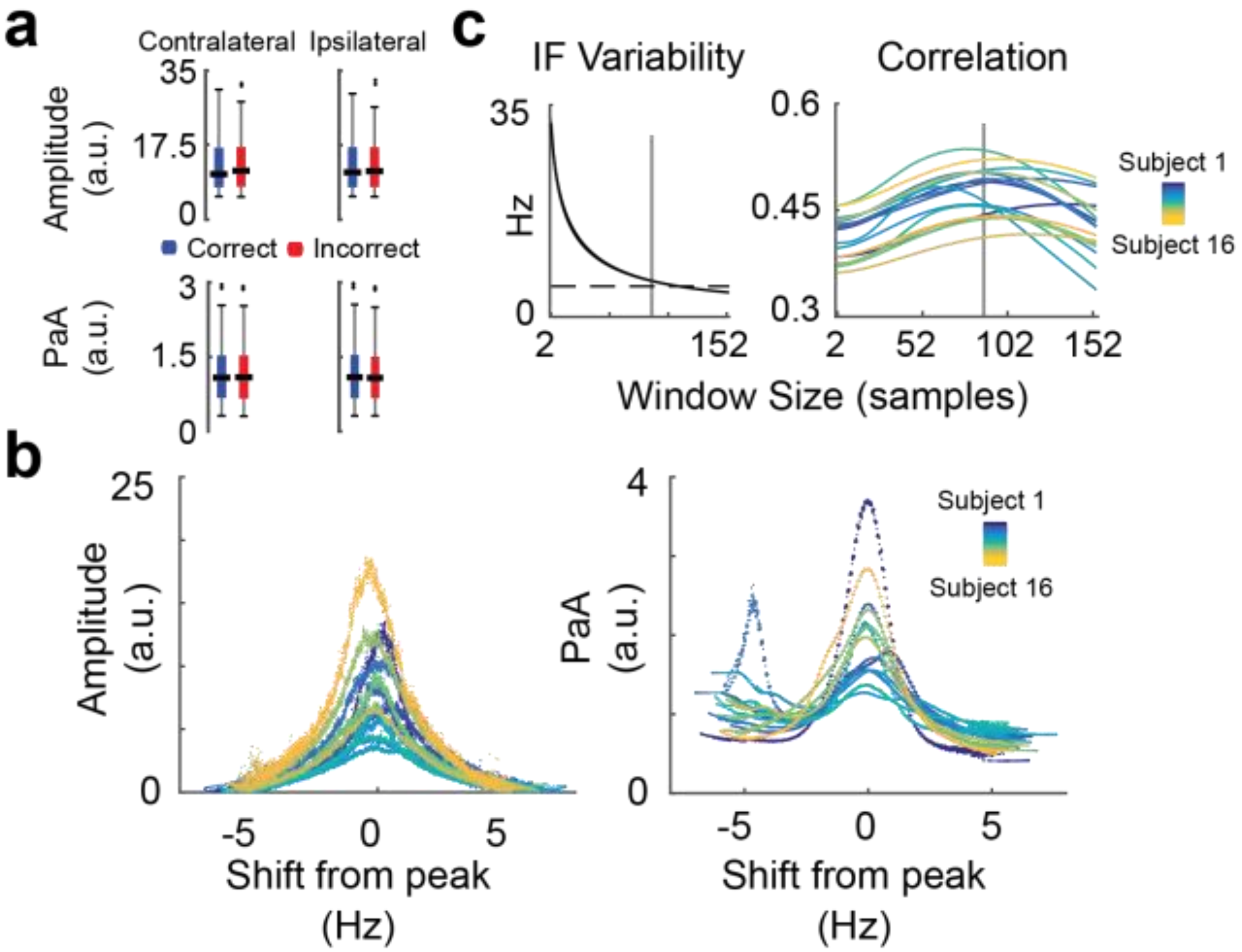

Supplementary Figure 2: Characterization of Instantaneous Frequency and PaA
a) The mean dynamic range of PaA and alpha amplitude on single trials. The max – min of PaA and Amplitude was taken on each trial, and the median over trials was stored for each subject and electrode. Data for all 3 contralateral electrodes were concatenated and displayed here. **b)** Mean amplitude and PaA vary with distance from peak alpha frequency. Each color is a single subject, all data are averaged over the 3 contralateral electrodes. Note that the shape of PaA reflects the amplitude spectra of each subject. **c)** PaA-Amplitude correlations and single trial instantaneous frequency dynamic range are plotted as a function of the window size used to estimate instantaneous frequency. The gray vertical line indicates the window size reported in previous analyses, chosen as the earliest point that variability nears the +/- 5 hz bandpass range.

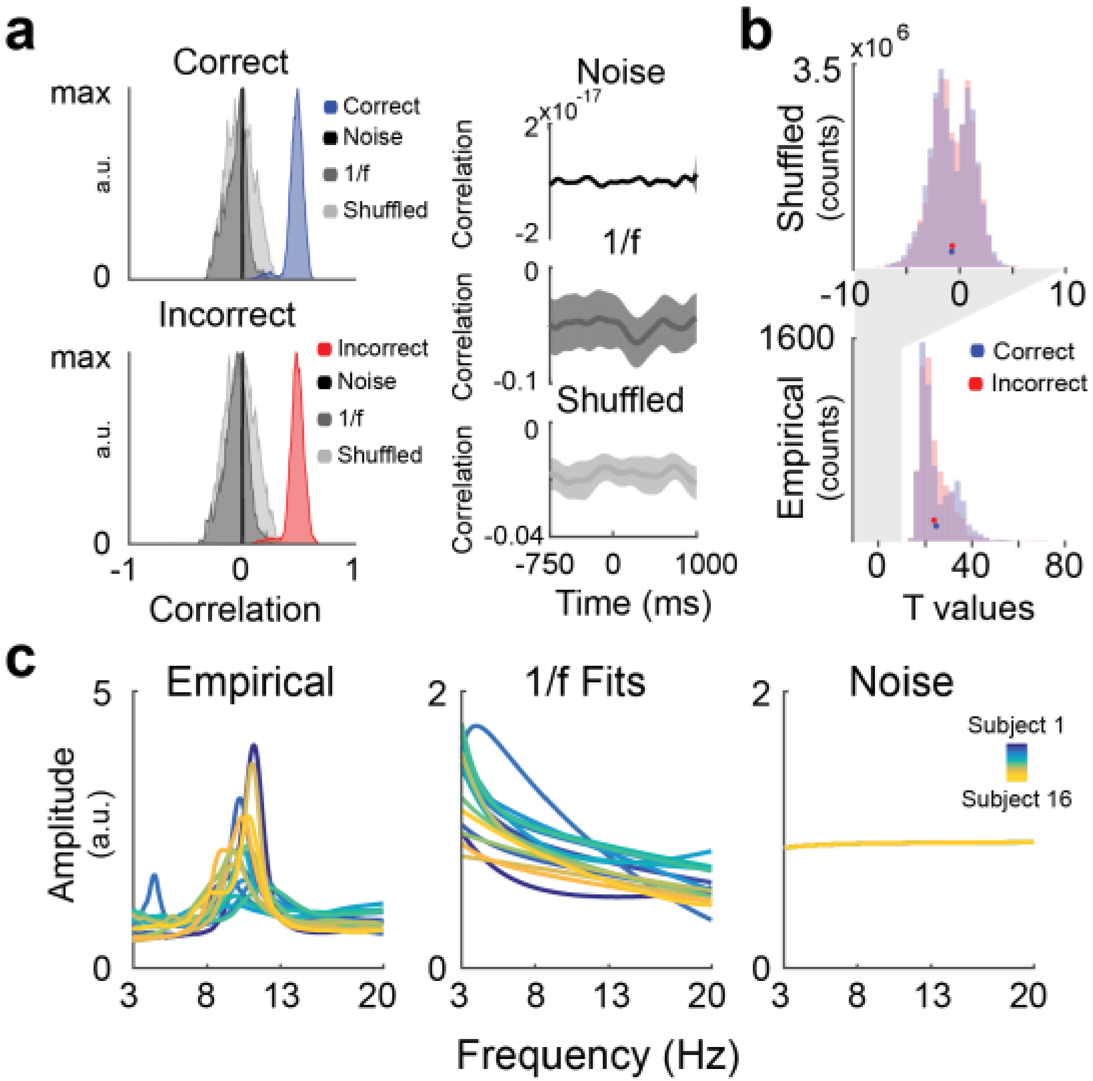

Supplementary Figure 3: Empirically observed correlations reflect an intrinsic relationship between amplitude and frequency

a) Plotted are histograms comparing the correlations for correct and incorrect trials for PaA computed with Noise, 1/f, and Shuffled LUTs (from black to light gray) with those empirically observed (blue and red) in contralateral electrodes. Each of these histograms are normalized to their maximum in order to show them on the same plot. The right panel shows timecourses for these alternative LUTs averaged over the contralateral electrodes. **b)** T-values computed from the shuffled spectrum are displayed for correct (blue) and incorrect (red) trials. Note the x-axis of these histograms correspond with the gray shaded area on the empirically observed T-value histogram. **c)** Empirical, 1/f fits and Noise LUTs averaged over contralateral electrodes are shown for each subject in a different color. Note the different y-axis for the empirical spectra. The “hooked” 1/f fit for the subject in blue is due to that subject’s additional prominent peak in the high delta/low theta range.

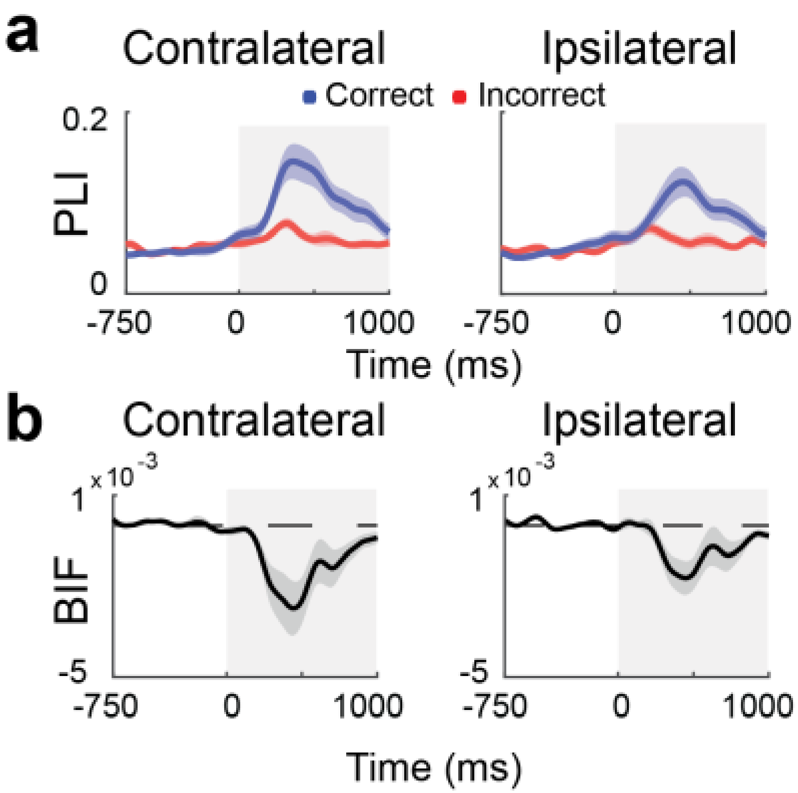

Supplementary Figure 4: Phase Locking and Bifurcation are not associated with accuracy

a) Contralateral and Ipsilateral phase locking values are plotted for correct and incorrect trials. There are no significant differences in prestimulus (indicated by the unshaded regions) phase locking between conditions. **b)** Similarly, prestimulus phase bifurcation (BIF) in contralateral and ipsilateral electrodes does not show significant difference from zero, indicating no significant phase concentration on either incorrect or correct trials (see Methods). Phase bifurcation ranges from 1 (perfect phase locking in both conditions at opposite phases) to -1 (perfect phase locking in only one condition), while values around zero indicate random distributions for correct and incorrect trials. Note that post-stimulus phase locking is not easily interpreted due to phase resets and ERPs[25,26,76].

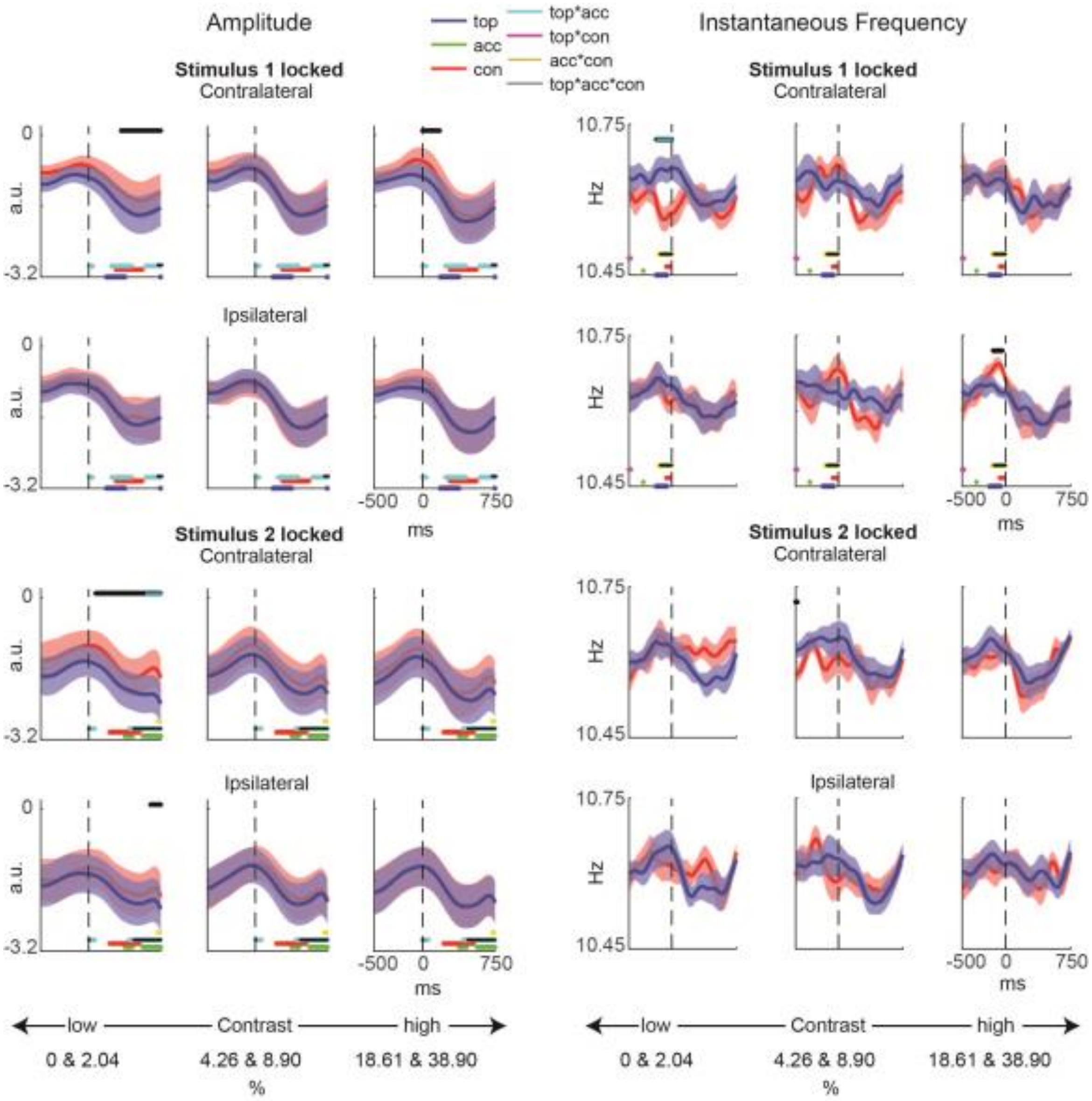

Supplementary Figure 5: Data from Experiment 2. Amplitude (left panel) and frequency (right panel) locked to the onset of the first stimulus (top half) and the onset of the second stimulus (bottom half). All plots are during the divided attention condition, and are shown as a function of topography (contralateral and ipsilateral) and behavioral performance (correct in blue, incorrect in red). Results of a 3-way repeated-measures ANOVA analysis with topography, accuracy and contrast level are plotted at the bottom of each subplot indicating uncorrected p-values < 0.05 with colors corresponding to the legend. Effects that survive FDR correction are in black (full results of this analysis are described in Supplementary Tables 5A and 5B). Post-hoc t-tests were performed between correct and incorrect timecourses to further understand these effects. Timepoints with $p < 0.05$ from these t-tests are plotted at the top of each subplot in black, with timepoints that survive FDR correction in cyan (contralateral) or yellow (ipsilateral). In keeping with previous figures, all amplitude statistics were performed on post-onset timepoints, while frequency statistics were performed on pre-onset timepoints. Note that like other analyses of Experiment 1, all p-values reported here were determined by randomizing conditions 5,000 times and comparing observed values to these empirical distributions.

	Correlation
**Contralateral vs Ipsilateral**	t(15) = 3.006, p=0.0072
**Contralateral Accuracy**	t(15) = -2.893, p=0.0015
**Ipsilateral Accuracy**	t(15) = -2.203, p=0.0467
**Location x Accuracy Interaction**	t(15) = 2.0355, p=0.0514

Supplementary Table 1: Predicted alpha amplitude – amplitude Correlations are stable. Predicted alpha amplitude shows a stable correlation with observed amplitude both over time and between experimental conditions. All tests report single time-point sliding t-tests performed on correlation values after values were compared against distributions obtained empirically by randomizing condition labels 10,000 times and then repeating the same statistical test (see Methods). Reported t-values are from the timepoint with smallest p-value. * indicates that p-values were significant after FDR correction at alpha = 0.05 from -500ms before stimulus onset to 1000ms after stimulus onset.

	Amplitude		PaA	
	**Prestimulus**	**Poststimulus**	**Prestimulus**	**Poststimulus**
**Frequency (bins 1-4)**	*F(3, 15) = 16.921, p=0.0	*F(3, 15) =16.895, p=0.0	*F(3,15) = 28.73, p = 0.0	*F(3, 15) =28.57, p=0.0
**Accuracy (correct vs incorrect)**	F(1, 15) = 0.12, p=0.748	*F(1, 15) = 9.169, p=0.0028	F(1, 15) = 0.229, p=0.669	F(1, 15) = 2.49, p=0.1328
**Frequency-Accuracy Interaction**	F(3, 15) = 0.11, p=0.958	F(3, 15) = 1.218, p=0.323	F(3, 15) = 0.269, p=0.858.	F(3, 15) = 0.314, p=0.829

Supplementary Table 2: Frequency and accuracy similarly predict amplitude and PaA. Within frequency and accuracy condition, mean amplitude and PaA in pre and post stimulus epochs from -350:-50 ms or 350:650 ms relative to the stimulus were averaged (timepoints chosen according to the significant timepoints from Figures 3 and 4). Trials were binned by their average pre or post stimulus frequency, and then amplitude and PaA was computed for each of these bins. We then computed a two-way repeated measures ANOVA with frequency and accuracy as factors. F-values were compared against distributions obtained empirically by randomizing condition labels 10,000 times and then repeating the same statistical test (see Methods). * indicates that p-values were significant alpha = 0.05. All statistics are reported for contralateral channels, as plotted in Figure 6C.

	Post Stimulus 1 Amplitude	Post Stimulus 2 Amplitude
**Topography (contralateral vs ipsilateral)**	$4.39 < F(1,14) < 6.73$<br>$0.02 \le p < 0.05$	n.s.
**Accuracy (correct vs incorrect)**	n.s.	$4.24 < F(1,14) < 5.60$<br>$0.026 \le p < 0.05$
**Contrast (levels 1-3)**	$3.23 < F(1,14) < 4.87$<br>$0.013 \le p < 0.05$	$3.19 < F(1,14) < 5.18$<br>$0.008 \le p < 0.05$
**Topography x Accuracy**	$4.46 < F(1,14) < 10.71$<br>*$0.005 \le p < 0.05$	$4.44 < F(1,14) < 15.40$<br>*$0 \le p < 0.05$
**Topography x Contrast**	n.s.	n.s.
**Accuracy x Contrast**	n.s.	$3.50 < F(1,14) < 3.65$<br>$0.047 \le p < 0.05$
**Topography x Accuracy x Contrast**	n.s.	n.s.

Supplementary Table 3: Amplitude during the divided attention condition as a function of electrode location and behavioral performance. All tests report the maximum and minimum timepoint-by-timepoint F-values over poststimulus timepoints. F-values were compared against distributions obtained empirically by randomizing condition labels 5,000 times and then repeating the same statistical test (see Methods). * indicates that p-values were significant after FDR correction at alpha = 0.05 from stimulus onset to +750ms. The minimum uncorrected p-value is also reported for each interval (note all intervals have a maximum uncorrected p-value of 0.05 since they were chosen on this basis).

	Pre Stimulus 1 Frequency	Pre Stimulus 2 Frequency
**Topography (contralateral vs ipsilateral)**	4.6 < F(1,14) < 8.44<br>0.009 ≤ p < 0.05	n.s.
**Accuracy (correct vs incorrect)**	4.41 < F(1,14) < 4.42<br>0.048 ≤ p < 0.05	n.s.
**Contrast (levels 1-3)**	3.31 < F(1,14) < 3.70<br>0.030 ≤ p < 0.05	n.s.
**Topography x Accuracy**	n.s.	n.s.
**Topography x Contrast**	3.37 < F(1,14) < 4.01<br>0.031 ≤ p < 0.05	n.s.
**Accuracy x Contrast**	3.31 < F(1,14) < 10.44<br>*0 ≤ p < 0.05	n.s.
**Topography x Accuracy x Contrast**	n.s.	n.s.

Supplementary Table 4: Frequency during the divided attention condition as a function of electrode location and behavioral performance. All tests report the maximum and minimum timepoint-by-timepoint F-values over prestimulus timepoints. F-values were compared against distributions obtained empirically by randomizing condition labels 5,000 times and then repeating the same statistical test (see Methods). * indicates that p-values were significant after FDR correction at alpha = 0.05 from -500 to stimulus onset. The minimum uncorrected p-value is also reported for each interval (note all intervals have a maximum uncorrected p-value of 0.05 since they were chosen on this basis).
